# Hospital Saturday and Sunday

**Published:** 1883-01

**Authors:** 


HOSPITAL SATURDAY AND SUNDAY.—It is rare that an imported custom, and that, too, an English one, should take such sudden and firm root in America as Hospital Saturday and Sunday in New-York. The setting apart of these last days in the year as days when collections in most of our churches and synagogues are made for the support of hospitals is a most praiseworthy act. It is impossible to give in detail the magnitude of the work performed by these worthy institutions ; enough to state that during last year 6,945 free patients received 261,705 days of hospital care gratuitously, at a cost of $330,517.55, in the several institutions forming the Hospital Saturday and Sunday Association. Of this large sum only $106,449.81 was received as income from their invested funds, while for the balance they are dependent upon the yearly gifts of the benevolent. In London, the contributions this year were upwards of a quarter of a million dollars. In our city it is noticeable how much greater is the proportion of donors among the poor than among the wealthy. The fact that the former may, and the latter probably never will, need the assistance of charity for restoration of health, may have something to do with this ; but we should be sorry to ascribe any but the most generous and unselfish motives to this large class of citizens in our city. The rich do not know how much is done in hospitals ; and hence it is a physician's duty to induce, as far as possible, the wealthy among his patients to give to a charitable fund. There is no medical man who harbors for a moment any but feelings of admiration for the hospitals and their work. In no wise do they detract from him, either pecuniarily or in reputation ; indeed, it is common for physicians themselves to send to these institutions those who for various reasons they cannot treat. Thus, a foul-smelling, close and dirty room, half warmed and filled with children, is but a sorry place for a weak woman to undergo an operation and in which to recover health and strength. It is the hospital where the physician can now send his patient, for philanthropical and interested as he may be, he could not personally shelter and treat one-hundredth part of the cases similar to this one that he meets with in his annual practice.

